# Biosynthesis of β-carotene in engineered E. coli using the MEP and MVA pathways

**DOI:** 10.1186/s12934-014-0160-x

**Published:** 2014-11-18

**Authors:** Jianming Yang, Lizhong Guo

**Affiliations:** Key Lab of Applied Mycology, College of Life Sciences, Qingdao Agricultural University, No.700 Changcheng Road, Chengyang District, Qingdao, 266109 China

**Keywords:** β-carotene, MEP pathway, MVA pathway, *E. coli*

## Abstract

**Background:**

β-carotene is a carotenoid compound that has been widely used not only in the industrial production of pharmaceuticals but also as nutraceuticals, animal feed additives, functional cosmetics, and food colorants. Currently, more than 90% of commercial β-carotene is produced by chemical synthesis. Due to the growing public concern over food safety, the use of chemically synthesized β-carotene as food additives or functional cosmetic agents has been severely controlled in recent years. This has reignited the enthusiasm for seeking natural β-carotene in large-scale fermentative production by microorganisms.

**Results:**

To increase β-carotene production by improving the isopentenyl pyrophosphate (IPP) and geranyl diphospate (GPP) concentration in the cell, the optimized MEP (methylerythritol 4-phosphate) pathway containing 1-deoxy-D-xylulose-5-phosphate synthase (DXS) and isopentenyl pyrophosphate isomerase (FNI) from *Bacillus subtilis*, geranyl diphosphate synthase (GPPS2) from *Abies grandis* have been co-expressed in an engineered *E. coli* strain. To further enhance the production of β-carotene, the hybrid MVA (mevalonate) pathway has been introduced into an engineered *E. coli* strain, co-expressed with the optimized MEP pathway and GPPS2. The final genetically modified strain, YJM49, can accumulate 122.4±6.2 mg/L β-carotene in flask culture, approximately 113-fold and 1.7 times greater than strain YJM39, which carries the native MEP pathway, and YJM45, which harbors the MVA pathway and the native MEP pathway, respectively. Subsequently, the fermentation process was optimized to enhance β-carotene production with a maximum titer of 256.8±10.4 mg/L. Finally, the fed-batch fermentation of β-carotene was evaluated using the optimized culture conditions. After induction for 56 h, the final engineered strain YJM49 accumulated 3.2 g/L β-carotene with a volumetric productivity of 0.37 mg/(L · h · OD_600_) in aerobic fed-batch fermentation, and the conversion efficiency of glycerol to β-carotene (gram to gram) reached 2.76%.

**Conclusions:**

In this paper, by using metabolic engineering techniques, the more efficient biosynthetic pathway of β-carotene was successfully assembled in *E. coli* BL21(DE3) with the optimized MEP (methylerythritol 4-phosphate) pathway, the gene for GPPS2 from *Abies grandis*, the hybrid MVA (mevalonate) pathway and β-carotene synthesis genes from *Erwinia herbicola*.

**Electronic supplementary material:**

The online version of this article (doi:10.1186/s12934-014-0160-x) contains supplementary material, which is available to authorized users.

## Background

β-carotene is a carotenoid compound that has been widely used not only in the industrial production of pharmaceuticals but also as nutraceuticals, animal feed additives, functional cosmetics, and food colorants [1]. Currently, more than 90% of commercial β-carotene is produced by chemical synthesis [2]. Due to the growing public concern over food safety, the use of chemically synthesized β-carotene as food additives or functional cosmetic agents has been severely controlled in recent years. This has reignited the enthusiasm for seeking natural β-carotene in large-scale fermentative production by *Blakeslea trispora*, a *Rhodotorula glutinis* mutant and the microalga *Dunaliella salina* [3-6].

Due to the availability of carotenoid genes from carotenogenic microbes, an alternative means of β-carotene production involves the heterologous expression of the β-carotene biosynthetic genes in non-carotenogenic microbes, e.g., *Escherichia coli*, *Zymomonas mobilis*, *Candida utilis*, and *Saccharomyces cerevisiae* [7]. Particularly, *E. coli* can serve as an excellent host for β-carotene production because of its powerful genetic tools for metabolic engineering and fast growth rate, and it has been successfully used in the production of various carotenoids, such as lycopene, astaxanthin and zeaxanthin [8-10].

With the rapid development of biocatalysis, β-carotene production through biosynthetic methods has become an active field, and several studies regarding genetic modification to enhance microbial production of β-carotene or carotenoids have been reported. Yoon S.H. *et al.* demonstrated that the production of 102 mg/L lycopene and 503 mg/L β-carotene was obtained from recombinant *E. coli* using the bottom portion of the MVA pathway of *Streptococcus pneumoniae* with exogenous supplementation of MVA [11]. To further improve the production of β-carotene, the whole MVA pathway and β-carotene synthesis genes were introduced into recombinant *E. coli* DH5α, and it produced 465 mg/L β-carotene at a glycerol concentration of 2% (w/v) [2]. This genetic modification was also performed on *Saccharomyces cerevisiae* harboring the native MVA pathway, which co-expressed the carotenogenic genes of *Xanthophyllomyces dendrorhous* with the mvaK1 (mevalonate kinase) gene from *Staphylococcus aureus* to enhance β-carotene production. This combination led to the production of 14.3 mg of β-carotene per liter in complex YPD medium [12]. Using a decentralized assembly strategy, a controllable β-carotene biosynthetic pathway was constructed in *Saccharomyces cerevisiae*, and the final engineered strain could accumulate 11 mg/g DCW of total carotenoids (72.57 mg/L) and 7.41 mg/g DCW of β-carotene in shaking flasks [13]. The above results also indicated that, as a host for β-carotene production, *E. coli* proves to be superior to *Saccharomyces cerevisiae*.

However, to date, the β-carotene yield remains unsatisfactory and unable to meet industrial demands. Therefore, further strain improvement, by metabolic engineering, is required. In this paper, the β-carotene metabolic pathway’s efficiency has been largely improved as follows. First, to increase β-carotene production by improving the IPP and GPP concentration in the cell, the optimized MEP pathway containing 1-deoxy-D-xylulose-5-phosphate synthase (DXS) and isopentenyl pyrophosphate isomerase (FNI) from *Bacillus subtilis*, geranyl diphosphate synthase (GPPS2) from *Abies grandis* were co-expressed in an engineered *E. coli* strain. Second, to further enhance the production of β-carotene, the hybrid MVA pathway [14] was introduced into an engineered *E. coli* strain, co-expressed with the optimized MEP pathway and geranyl diphosphate synthase (GPPS2). The final genetically modified strain, YJM49, could accumulate 122.4±6.2 mg/L β-carotene in flask culture, approximately 113 and 1.7 times greater than strain YJM39, which carries the native MEP pathway, and YJM45, which harbors the MVA pathway and the native MEP pathway, respectively. After culture condition optimization, the final engineered strain could accumulate 256.8±10.4 mg/L and 3.2 g/L β-carotene under flask cultivation and fed-batch fermentation conditions, respectively.

## Results and discussion

### The biosynthesis of β-carotene using the optimized MEP pathway in engineered *E. coli*

**Figure 1:**
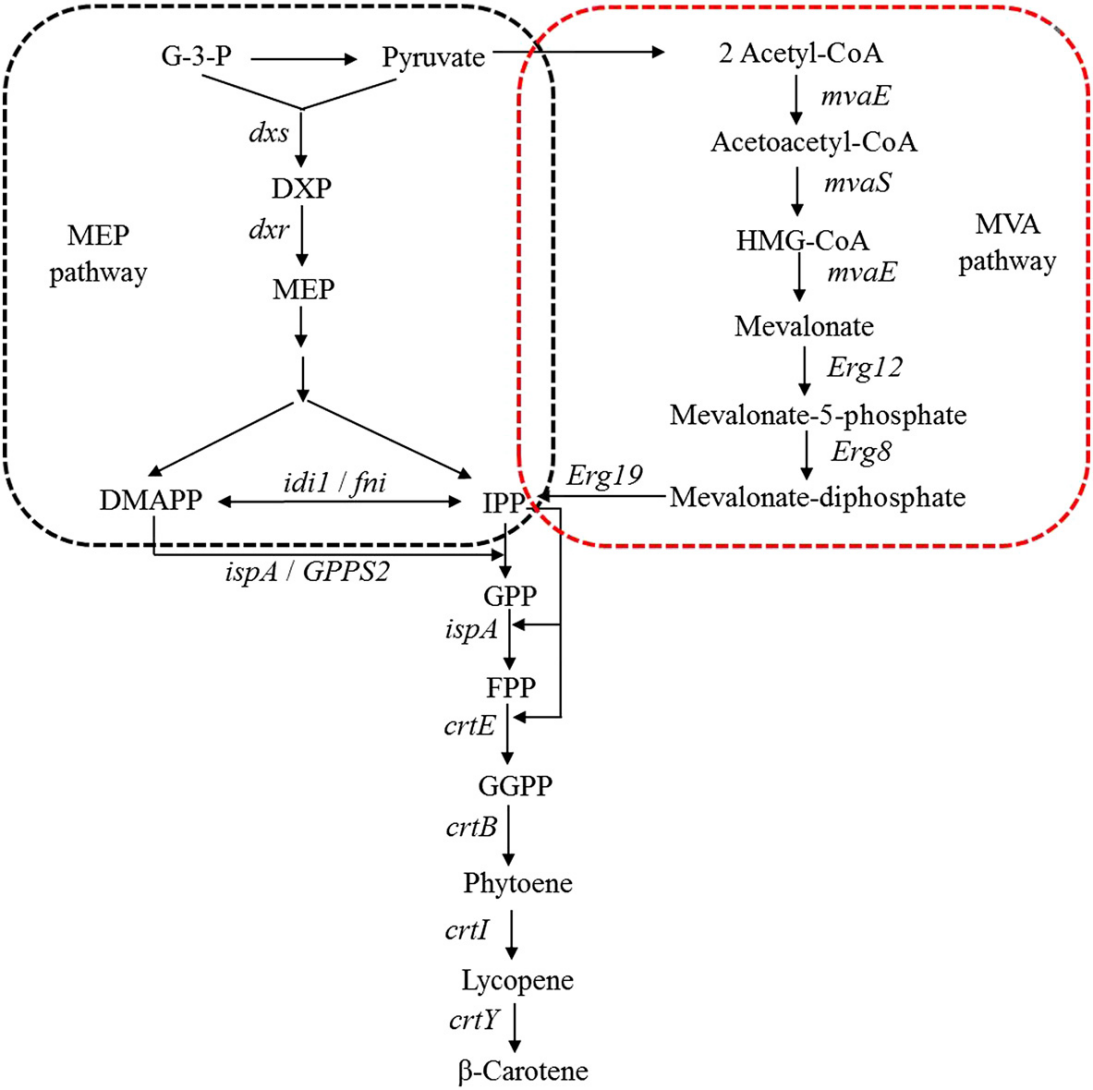
**Biosynthesis of β-carotene via both the MEP and MVA pathways used in this study.** DMAPP and IPP, precursors for β-carotene biosynthesis are synthesized via both the MEP and MVA pathways. The MVA pathway was divided into two portions, the upper (acetyl-CoA to MVA) and lower (MVA to IPP and DMAPP). The upper portion is composed of the *mvaE* (acetyl-CoA acetyltransferase), *mvaS* (HMG-CoA synthase) and *mvaE* (HMG-CoA reductase) genes, which are from *E. faecalis*, while the lower is composed of the *Erg12* (mevalonate kinase), *Erg8* (phosphomevalonate kinase), *Erg19* (diphosphomevalonate decarboxylase) and *idi1* (IPP isomerase) genes, which are from *S. cerevisiae*. DMAPP and IPP are converted to GPP by IspA (GPP/FPP synthase) from *E. coli* and by GPPS2 (GPP synthase) from *A. grandis*. FPP is transformed into β-carotene via the foreign carotenoid synthesis pathway which includes *crtE* (GGPP synthase), *crtB* (phytoene synthase), *crtI* (phytoene desaturase) and *crtY* (lycopene cyclase) from *E. herbicola. dxs* (DXP synthase) and *fni* (IPP isomerase) genes from *B. subtilis*. Abbreviations: G-3-P, glyceraldehyde-3-phosphate; DXP, 1-deoxy-d-xylulose-5-phosphate; MEP, 2-C-methyl-d-erythritol-4-phosphate; HMG-CoA, 3-hydroxy-3-methylglutaryl-CoA; IPP, isopentenyl diphosphate; DMAPP, dimethylallyl diphosphate; GPP, geranyl diphosphate; FPP, farnesyl diphosphate; GGPP, geranylgeranyl diphosphate.

**Figure 2:**
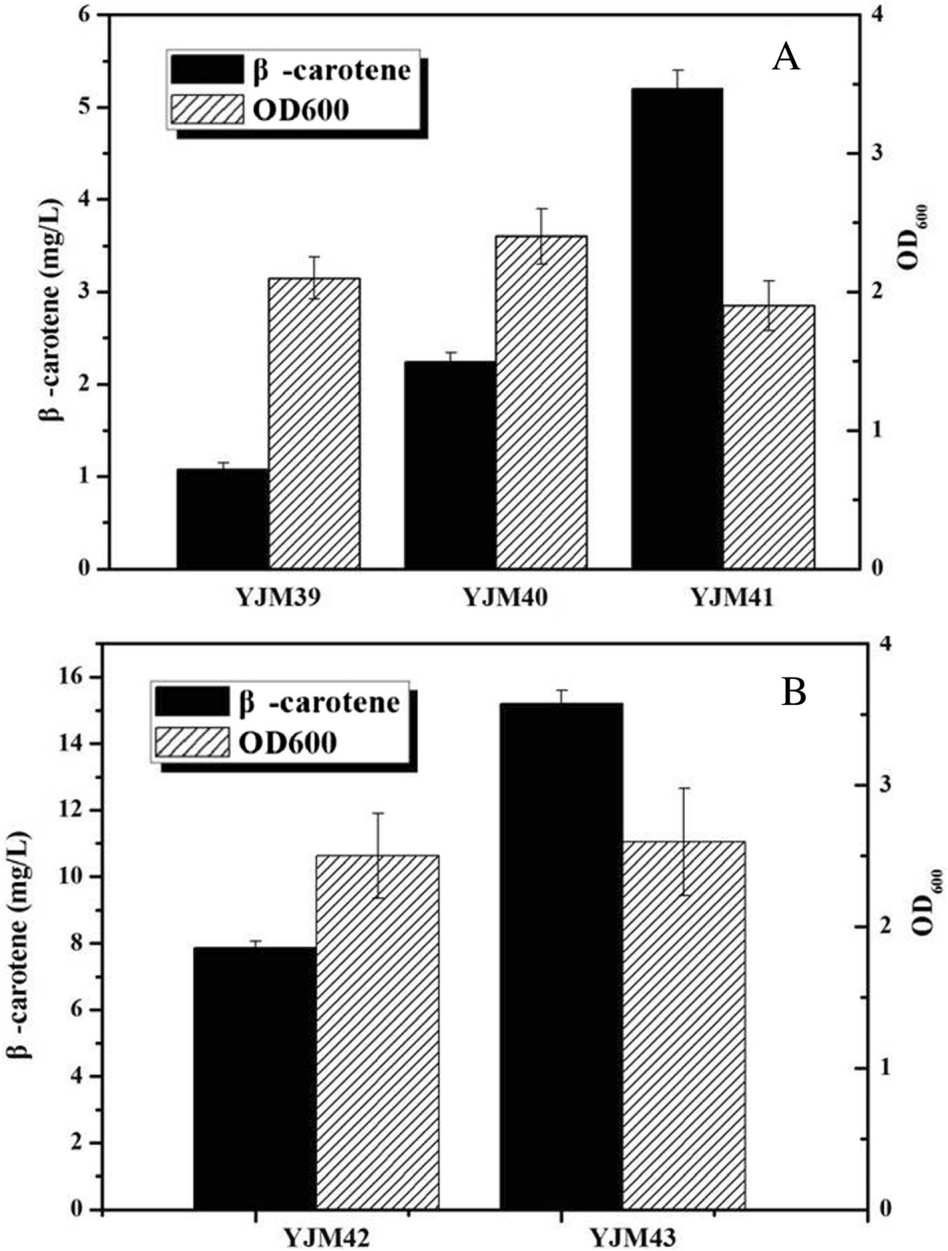
**The effect of enzymes from the MEP pathway and GPP synthase on β-carotene production. A**: The effect of enzymes from MEP pathway on β-carotene production. YJM40 containing the DXS enzyme from *B. subtilis*, YJM41 containing DXS and FNI enzymes from *Bacillus subtilis*; **B**: The effect of GPP synthase on β-carotene production. YJM42 harboring IspA from *E. coli*, YJM43 bearing GPPS2 from *A. grandis*. All the experiments were carried out in triplicates.

All carotenoids, including β-carotene, are produced via the common precursors isopentenyl diphosphate (IPP) and dimethylallyl diphosphate (DMAPP), which are synthesized through either the well-characterized mevalonate (MVA) pathway or the recently discovered non-mevalonate pathway (MEP) [15] (Figure 1). One of the rate-controlling steps in the heterogeneous carotenoid biosynthesis is the supply of IPP and DMAPP [16-18]. In a previous study, DXS enzyme was proven to be the first rate-limiting step of the MEP pathway, and overexpression of DXS enzyme could improve the production of isoprenoid [19-21]. Hence, to enhance the supply of IPP and DMAPP for the production of β-carotene, the heterologous *dxs* gene from *Bacillus subtilis* was overexpressed in *E. coli* BL21(DE3) containing the plasmid pAC-BETA. As shown in Figure 2A, a noticeable difference in β-carotene production was observed. The *E. coli* strain YJM40 (*E. coli* BL21(DE3)/pAC-BETA, pYJM40) produced 2.24±0.1 mg of β-carotene per liter of bacterial culture, which was approximately 2-fold higher than that produced by the strain YJM39 (1.08±0.07 mg/L, *E. coli* BL21(DE3)/pAC-BETA), and the *E. coli* strain carrying no plasmid generated no detectable β-carotene.

Isopentenyl diphosphate (IPP) isomerase catalyzes the reversible interconversion of IPP to DMAPP and possesses two types of IPP isomerase that show no similarity in their amino acid sequences [22,23]. Type I IPP isomerase (IDI-1) utilizes a divalent metal in a protonation-deprotonation reaction, whereas type II IPP isomerase (IDI-2) requires reduced flavin [24]. In previous reports, type II IDI was shown to perform more efficiently for isoprenoid production than type I IDI [22,25]. Therefore, to further increase the production of β-carotene, the FNI enzyme (isopentenyl diphosphate isomerase), a type II IPP isomerase from *Bacillus subtilis*, was overexpressed in YJM40 (*E. coli* BL21(DE3)/pAC-BETA, pYJM40). As shown in Figure 2A, the strain YJM41, containing plasmids pYJM41 and pACY-BETA could accumulate 5.2±0.2 mg/L β-carotene, which was 4.8 and 2.3 times greater than the levels produced by YJM39 and YJM40, respectively. This result demonstrated that the exogenous expression of the DXS and FNI enzymes contributed to the increased β-carotene production. Thus, the biosynthetic pathway for β-carotene production was successfully constructed using the native MEP pathway, the foreign DXS and FNI enzyme from *Bacillus subtilis* and β-carotene synthesis genes from *Erwinia herbicola*.

### The effect of the GPPS enzyme on the production of β-carotene using the MEP pathway

Geranyl diphosphate (GPP), which is an important metabolic precursor of carotenoids, is derived from the condensation of dimethylallyl diphosphate and isopentenyl diphosphate and is catalyzed by GPP synthase [26]. In this paper, the GPP enzymes of *Abies grandis* and *E. coli* were tested for their ability to enhance the supply of GPP and increase β-carotene production.

The *IspA* gene from *E. coli* and the *GPPS2* gene from *A. grandis* were cloned into the plasmid pETDuet-1 along with the *dxs* and *fni* genes from *B. subtilis* to create the plasmids pYJM42 or pYJM43, respectively. These plasmids were subsequently introduced into *E. coli* BL21(DE3)/pAC-BETA to screen for the GPP synthase. The strains YJM42 and YJM43 were cultured in 500-ml shake-flasks with 200 mL fermentation medium. When each culture reached an OD_600_ of 0.6-0.9, expression of the heterologous genes was induced with 0.25 mM IPTG, and the culture was further incubated at 30°C for 24 h. A significant difference in the β-carotene concentration was found between the two strains. The strain YJM43 produced 15.2±0.4 mg/L β-carotene, while the strain YJM42 produced 7.86±0.2 mg/L (Figure 2B). This result demonstrates that the exogenous expression of GPPS contributed to the β-carotene production, and the GPPS enzyme from *A. grandis* (GPPS2) is more efficient than the one from *E. coli* (IspA) for producing higher levels of β-carotene, which was in line with the production of other terpenes such as α-pinene and sabinene in previous studies [27,28].

### The biosynthesis of β-carotene using the MVA pathway in engineered *E. coli*

Although much progress in β-carotene production using the MEP pathway has been made, this approach is still suffering from limitations due to the regulatory mechanisms present in the native host [29]. To circumvent the native limitations of the MEP pathway, a hybrid exogenous MVA pathway [14], which was proved to be effective in supplying the precursors DMAPP and IPP, was introduced into *E. coli* to enhance the production of β-carotene.

**Figure 3:**
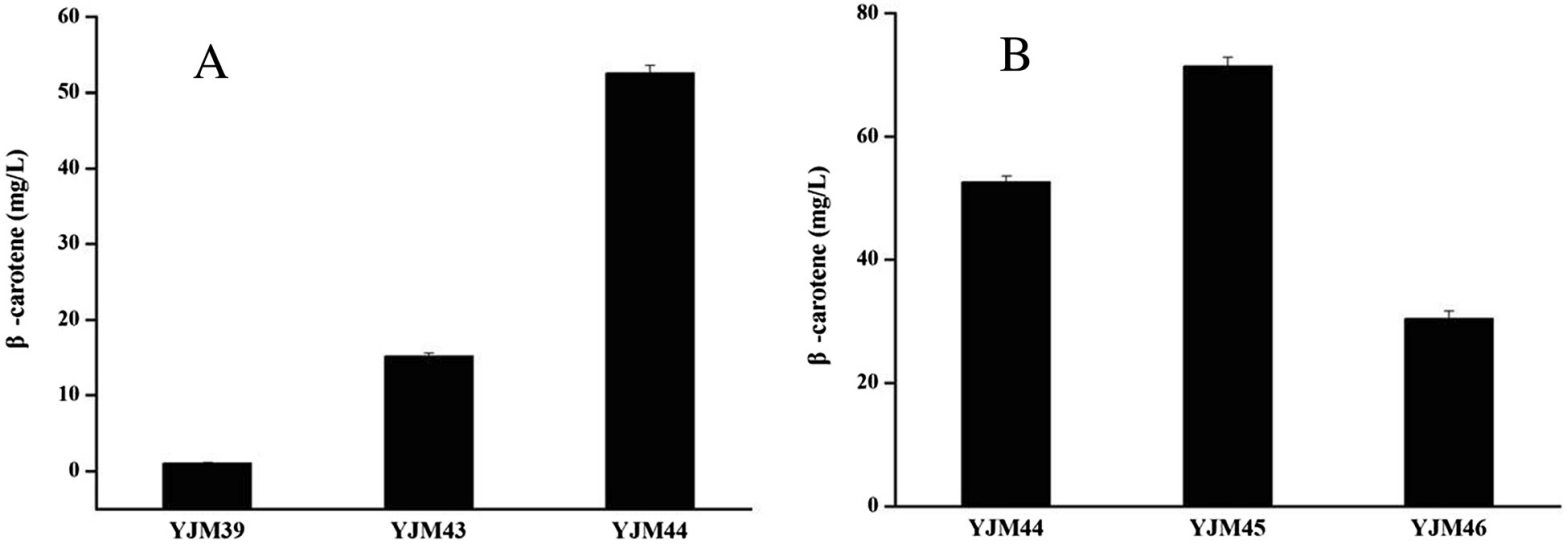
**The effect of the metabolic pathway and expression level of the MVA upper pathway on β-carotene production. A**: The effect of metabolic pathway on β-carotene production. The pathway details were described in Figure 1. The β-carotene titer of strain YJM44 harboring the hybrid MVA pathway reached 52.6±1.5 mg/L, which was approximately 49-fold and 3.5-fold higher than that of strains YJM39, which bears the native MEP pathway, and YJM43, which carries the optimized MEP pathway with the overexpression of the DXS and FNI enzymes from *B. subtilis* and the GPPS2 enzyme from *A. grandis*, respectively. **B**: The effect of expression level of the MVA upper pathway on β-carotene production. The MVA upper pathway under the control of the T7 promoter (YJM44) achieved much higher β-carotene production than it did under the control of the *ara*BAD promoter (YJM46). The strain YJM45, using a low copy number plasmid, reached the highest β-carotene production (71.4±4.3 mg/L). The experiment was conducted in triplicate.

To verify the effect of the hybrid MVA pathway on the production of β-carotene, the recombinant strains YJM44 (*E. coli* BL21(DE3) harboring the hybrid MVA pathway, pAC-BETA), YJM43 (*E. coli* BL21(DE3) carrying the optimized MEP pathway and GPPS2, pAC-BETA) and YJM39 (*E. coli* bearing the native MEP pathway, pAC-BETA) were cultured in fermentation medium under shake-flask conditions. The β-carotene titer of strain YJM44 reached 52.6±1.5 mg/L after being induced by 0.25 mM IPTG for 24 h (Figure 3A). The titer was approximately 49-fold and 3.5-fold higher than that of the strains YJM39 and YJM43, respectively, when cultured under the same conditions. These results suggest that the hybrid MVA pathway resulted in a huge increase in β-carotene production.

To further enhance β-carotene production, the expression level of the MVA upper pathway genes from *E. faecalis* was optimized using different plasmid vectors, which contained different copy numbers and promoters. As shown in Figure 3B, the MVA upper pathway genes achieved much higher β-carotene production when under the control of the T7 promoter (YJM44) instead of the *ara*BAD promoter (YJM46). Using a low copy number plasmid, strain YJM45 achieved the highest β-carotene production (71.4±4.3 mg/L), which was 1.36-fold higher than the level produced by YJM44 using a high copy number plasmid.

### The biosynthesis of β-carotene using the MVA and MEP pathways in engineered *E. coli*

To improve β-carotene production, the optimized MEP and MVA pathways were co-expressed in *E. coli*. The *fni*, *dxs* and *GPPS2* genes were introduced into the pCol-mvaE-mvaS plasmid to form pYJM49. Then, plasmid pYJM49 was co-expressed in *E. coli* BL21(DE3) harboring the plasmids pYJM14 and pAC-BETA to create strain YJM49. The strain YJM49 was cultured in 500-ml shake-flasks. When the culture reached an OD_600_ of 0.6, expression of the MEP and MVA pathway genes was induced with 0.25 mM IPTG, and the culture was further incubated at 30°C for 24 h. As shown in Table 1, the *E. coli* strain YJM49 produced 122.4±6.2 mg of β-carotene per liter of bacterial culture, which was approximately 113-fold and 1.7-fold higher than that produced by strains YJM39 (1.08±0.07 mg/L), which carried the native MEP pathway, and YJM45 (71.4±4.3 mg/L), which harbors the MVA pathway and the native MEP pathway, respectively. This result indicated that co-expression of the optimized MEP and MVA pathways in engineered *E. coli* could result in a considerable increase in β-carotene production.

**Table 1:** **β-carotene production by** ***E. coli*** **strains harboring different metabolic pathways under flask conditions**

**Strains (pathway)**	**β-Carotene (mg/L)**	**Fold**
YJM49 (MVA+the optimized MEP+GPPS2)	122.4±6.2	113
YJM45 (MVA+the native MEP)	71.4±4.3	66
YJM39 (the native MEP)	1.08±0.07	1

The experiment was performed in triplicate.

### Optimization of culture conditions

Optimization of culture conditions is a useful method to enhance quality and quantity of β-carotene production. The culture conditions that can be optimized are the nitrogen source, which can provide trace nutrition for microorganisms [30], the type of carbon source, which can affect the concentration of intracellular acetyl-CoA (a starting metabolite for carotenoid synthesis) [31], the level of IPTG, which can adjust the extent of the metabolic burden imposed on the cell [32], and the cultivation temperature, which can balance enzyme expression, cell growth and product formation [33].

**Figure 4:**
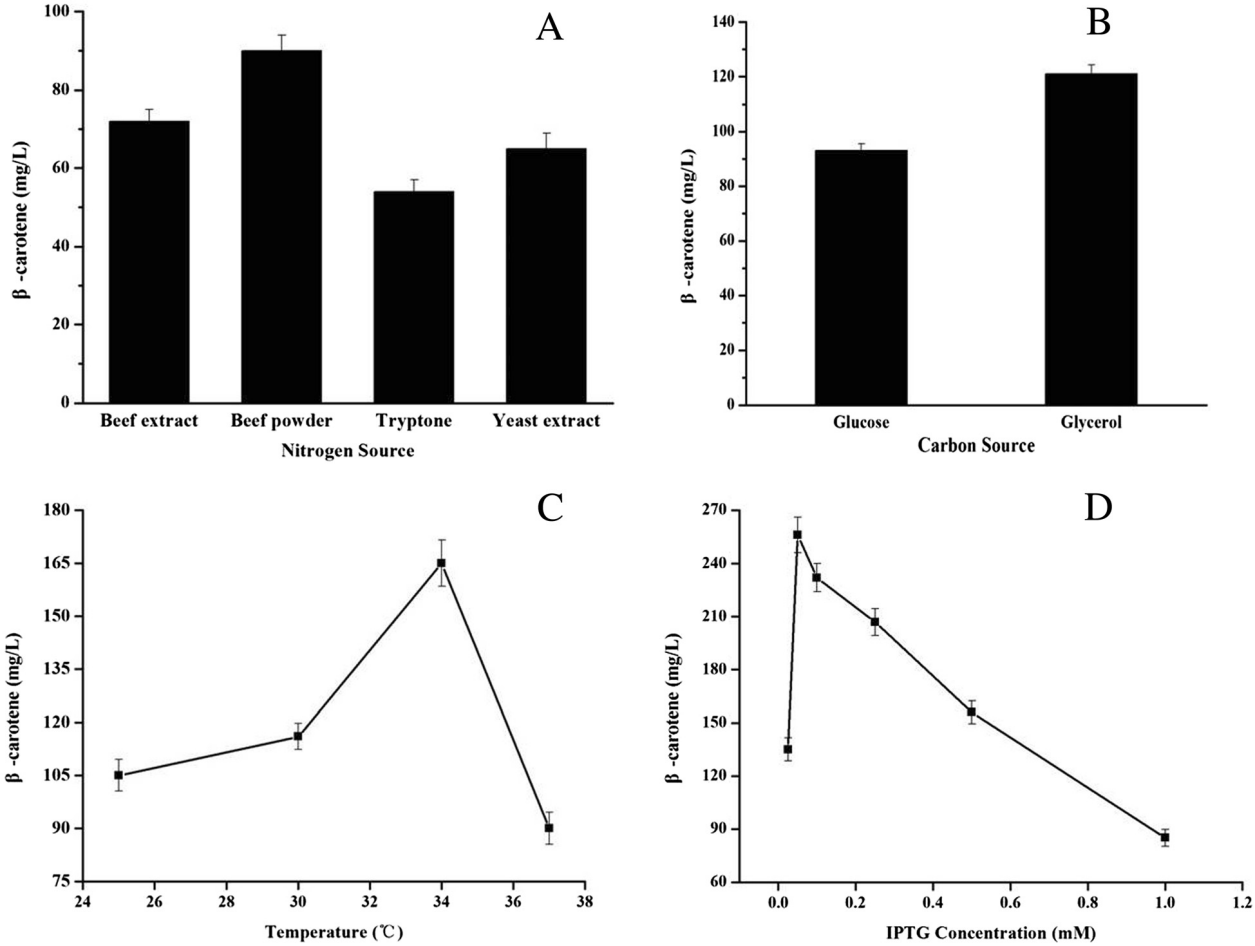
**The effects of fermentation source and culture conditions on β-carotene production by YJM49. A**: The effect of nitrogen sources on β-carotene production. **B**: The effect of carbon sources on β-carotene production. **C**: The effect of induction temperatures on β-carotene production. **D**: The effect of inducer concentration on β-carotene production. When OD_600_ reached 0.6-0.9, cultures were induced for 48 h using IPTG in shake-flasks. All the experiments were carried out in triplicates. Optimized conditions: Nitrogen sources, beef power; Carbon source, glycerol; Temperature, 34°C; IPTG concentration, 0.05 mM.

In this study, to improve β-carotene yield, the organic nitrogen source, carbon source, induction temperature and IPTG concentration were optimized using the “one-factor at-a-time” optimization strategy (Figure 4). The highest β-carotene production (256.8±10.4 mg/L) was achieved when the YJM49 strain was cultivated in fermentation medium containing 20 g/L glycerol as a carbon source, 10 g/L beef powder as an organic nitrogen source and induced with 0.05 mM IPTG at 34°C. This optimization resulted in an approximately 2-fold increase in β-carotene production.

In this paper, using glycerol as a carbon source greatly improved β-carotene production, which may be a result of a higher acetate concentration in the cultures containing glucose than in the cultures containing glycerol. The high concentration of acetate acts as an inhibitory metabolite, lowering β-carotene production. Meanwhile, glucose, a readily metabolizable carbon source, has been reported to catabolically repress the T7 promoter for the whole MVA pathway and the *trc* promoter for the β-carotene synthesis pathway [34].

### Fed-batch fermentation

**Figure 5:**
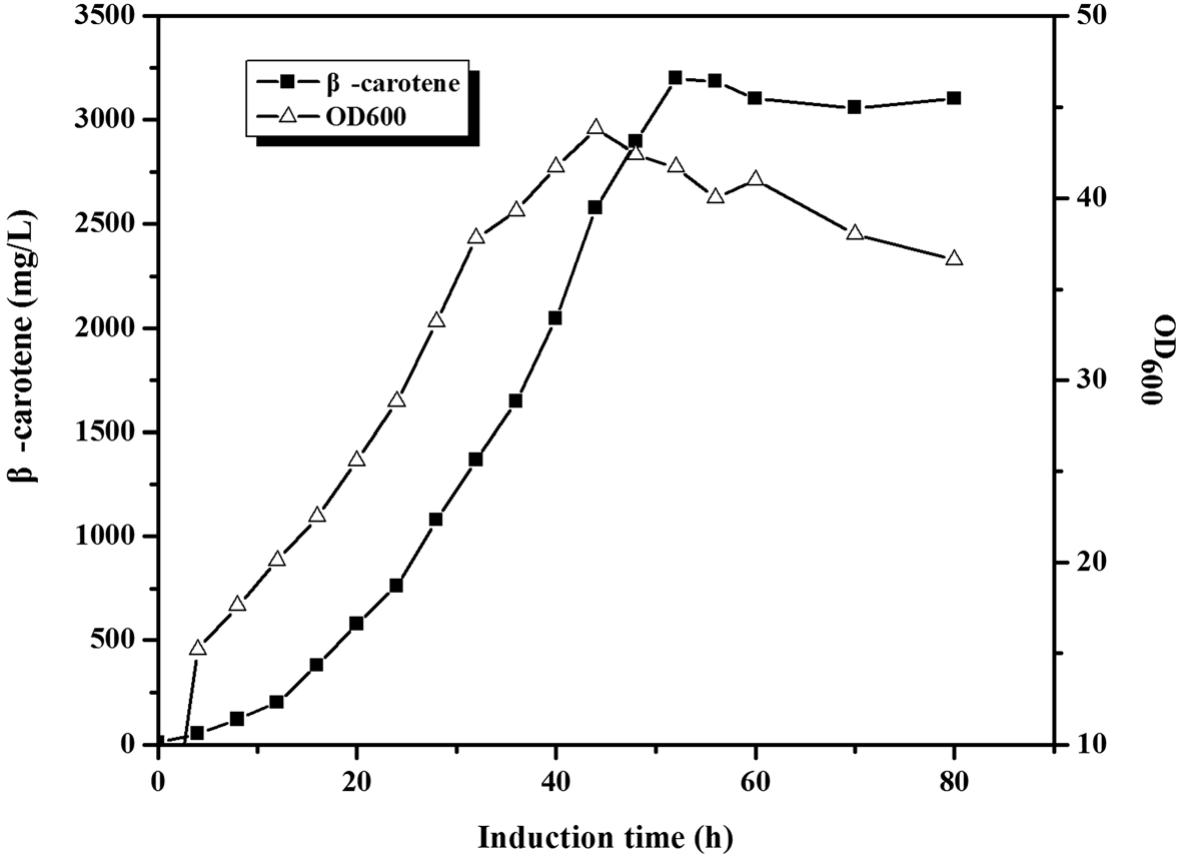
**The time course of β-carotene production by YJM49.** β-carotene accumulation(■)and cell growth (Δ) in YJM49, Induction was carried out at an OD_600_ of 12. Other experimental conditions were described in ‘Fed-batch fermentation’.

We performed the fed-batch fermentation based on the results obtained with the flask cultures. The fermentations were conducted under aerobic condition using YJM49 and a fermentation medium containing glycerol as above. Glycerol was added continuously when the initial carbon source was exhausted which was indicated by the sharp rise of DO. Cell density and β-carotene accumulation were monitored over the course of the fermentation (Figure 5). Figure 5 shows the time profile for the cell density and β-carotene production for 80-h fed-batch fermentations. For approximately 44 h post-induction, the bacteria grew quickly to an OD_600_ of 40, and the titer of β-carotene increased gradually along with the bacterial cell growth. The highest β-carotene production, 3.2 g/L, was obtained after induction for 52 h. This production rate corresponds to a volumetric productivity of 0.37 mg/(L · h · OD_600_), and the conversion efficiency of glycerol to β-carotene (gram to gram) reached 2.76%. The above results obtained under the fermentor level demonstrated that this engineered *E. coli* strain had the potential to produce β-carotene on a large scale.

Compared with the β-carotene pathway constructed by Yoon S.H. *et al*. [2], the optimized MEP pathway containing *dxs* and *fni* genes from *B. subtilis* and the *GPPS2* gene from *A. grandis* were used in our engineered strain. The *dxs* and *fni* genes were used to increase the IPP and DMAPP content, and the overexpression of the *GPPS2* gene improved the GPP concentration. Our experimental data showed that increasing the cellular concentration of IPP, DMAPP and GPP could lead to an increase in β-carotene accumulation.

Despite the progress already achieved in microbial β-carotene production, some problems still remained unsolved. One problem is the retardation of cell growth resulting from the overexpression of many heterologous genes. This problem could be solved by employing a chromosome integration technique to decrease the cell growth burden on the host, which results from the overexpression of heterologous genes [35,36]. Another problem is how to regulate suitable expression levels for many of the genes in the metabolic pathway. A possible solution is to seek the natural sensor for the key intermediate of MVA or other metabolic products, and then to develop a dynamic sensor-regulator system (DSRS), which could dynamically regulate the expression of genes involved in target product synthesis and balance metabolism [37]. Finally, the supply of NADPH is insufficient in the cell may limit the synthesis of the end product, which can be solved by activating the pentose phosphate pathway (PPP) and Entner-Doudoroff pathway (ED) [38].

## Conclusions

In this study, we improved microbial β-carotene production from glycerol by using the optimized MEP and MVA pathways and the culture condition optimization. When *dxs*, *fni*, *GPPS2*, and the whole MVA pathway were co-expressed, the final engineered YJM49 strain accumulated 3.2 g/L β-carotene from glycerol in an aerobic fed-batch fermentation, and the conversion efficiency of glycerol to β-carotene (gram to gram) reached 2.76%. This is the highest level of β-carotene produced from inexpensive carbon sources yet reported.

## Methods

### Bacterial strains, plasmids, and growth conditions

All strains and plasmids used in this study are listed in Table 2. *E. coli* BL21(DE3) (Invitrogen, Carlsbad, CA) was used as the host to overexpress proteins and produce β-carotene. Cultures were grown aerobically at 37°C in Luria Broth (per liter: 10 g Difco tryptone, 5 g Difco yeast extract and 5 g NaCl). For β-carotene production, recombinant strains were cultured in shake-flask or fed-batch fermentation with the initial fermentation medium containing glucose 20 g/L, K_2_HPO_4_ 9.8 g/L, beef extract 5 g/L, ferric ammonium citrate 0.3 g/L, citric acid monohydrate 2.1 g/L, MgSO_4_ 0.06 g/L and 1 mL trace element solution which includes (NH_4_)_6_Mo_7_O_24_ · 4H_2_O 0.37 g/L, ZnSO_4_ · 7H_2_O 0.29 g/L, H_3_BO_4_ 2.47 g/L, CuSO_4_ · 5H_2_O 0.25 g/L, and MnCl_2_ · 4H_2_O 1.58 g/L. If necessary, appropriate antibiotics were added to the culture medium at the following concentration: ampicillin (Amp, 100 μg/mL), kanamycin (Kan, 50 μg/mL), and chloramphenicol (Cm, 34 μg/mL).

**Table 2:** **Strains and plasmids used in this study**

**Name**	**Relevant characteristics**	**References**
**Strains**		
*E.coli* BL21(DE3)	F^−^ *ompT hsd*S_B_ (r_B_^−^m_B_^−^) *gal dcm rne*131 λ(DE3)	Invitrogen
*E.coli* DH5α	*deoR*, recA1, endA1, hsdR17(rk-,mk+), phoA, supE44, λ-, thi-1, gyrA96, relA1	Takara
YJM39	*E.coli* BL21(DE3)/pAC-BETA	This study
YJM40	*E.coli* BL21(DE3)/pYJM40, pAC-BETA	This study
YJM41	*E.coli* BL21(DE3)/pYJM41, pAC-BETA	This study
YJM42	*E.coli* BL21(DE3)/pYJM42, pAC-BETA	This study
YJM43	*E.coli* BL21(DE3)/pYJM43, pAC-BETA	This study
YJM44	*E.coli* BL21(DE3)/pYJM44, pYJM14, pAC-BETA	This study
YJM45	*E.coli* BL21(DE3)/pYJM45, pYJM14, pAC-BETA	This study
YJM46	*E.coli* BL21(DE3)/pYJM46, pYJM14, pAC-BETA	This study
YJM49	*E.coli* BL21(DE3)/pYJM49, pYJM14, pAC-BETA	This study
**Plasmids**		
pETDuet-1	pBR322 *ori*, *lacI* T7*lac*, Amp^r^	Novagen
pET-28a(+)	pBR322 *ori*, *lacI* T7*lac*, Kan^r^	Novagen
pCOLADuet-1	ColA *ori*, *lacI* T7*lac*, Kan^r^	Novagen
pBAD 18	pBR322 *ori*, *ara*BAD, Amp^r^	[34]
pTrcHis2B	pBR322 origin, Amp^r^	Invitrogen
pYJM14	pTrcHis2B carrying *ERG12*, *ERG8*, *ERG19* and *IDI1* from *Saccharomyces cerevisiae*	[39]
pAC-BETA	pACYC184 carrying *crtE*, *crtB*, *crtI* and *crtY* from *Erwinia herbicola*	[40]
pYJM40	pETDuet-1 carrying *dxs* from *Bacillus subtilis*	This study
pYJM41	pETDuet-1 carrying *dxs* and *fni* from *Bacillus subtilis*	This study
pYJM42	pETDuet-1 carrying *dxs* and *fni* from *Bacillus subtilis*, *ispA* from *E.coli* BL21(DE3)	This study
pYJM43	pETDuet-1 carrying *dxs* and *fni* from *Bacillus subtilis*, *GPPS2* from *Abies grandis*	This study
pYJM44	pET-28a(+) carrying *mvaE and mvaS* from *Enterococcus faecalis*	This study
pYJM45	pCOLADuet-1 carrying *mvaE and mvaS* from *Enterococcus faecalis*	This study
pYJM46	pBAD18 carrying *mvaE and mvaS* from *Enterococcus faecalis*	This study
pYJM47	pCOLADuet-1 carrying *mvaE and mvaS* from *Enterococcus faecalis*, *fni* from *Bacillus subtilis*	This study
pYJM48	pCOLADuet-1 carrying *mvaE and mvaS* from *Enterococcus faecalis*, *fni* from *Bacillus subtilis*, *GPPS2* from *Abies grandis*	This study
pYJM49	pCOLADuet-1 carrying *mvaE and mvaS* from *Enterococcus faecalis*, *dxs* and *fni* from *Bacillus subtilis*, *GPPS2* from *Abies grandis*	This study

### Plasmid construction

Common procedures were performed according to standard protocols of Sambrook *et al*. [41]. Polymerase chain reaction (PCR) was carried out using *Pfu* DNA polymerase (TaKaRa, Dalian, China) following the manufacturer’s instructions.

### Construction of the plasmids for the optimized MEP pathway of β-carotene synthesis

The *dxs* gene was obtained by PCR using the primers dxs-F (5′-CATGCCATGGGCGATCTTTTATCAATACAGGACC-3′) and dxs-R (5′- CGCGGATCCTCATGATCCAATTCCTTTGTGT-3′) and *Bacillus subtilis* genomic DNA as a template. The isolated *dxs* gene fragment was excised using NcoI and BamHI, followed by insertion into the corresponding sites in the vector pETDuet-1 to create pYJM40. The *fni* gene was obtained by PCR using the primers fni-F (5′-GGAAGATCT CACTCGAGCAGAACGAAA AAGA-3′) and fni-R (5′-C GGGGTACCTTATCGCACACTATAGCTTGA-3′) and *Bacillus subtilis* genomic DNA as a template. The isolated *fni* gene fragment was excised using BglII and KpnI, followed by insertion into the corresponding sites of the vector pYJM40 to create pYJM41.

The *IspA* gene was obtained by PCR using the primers IspA-F (5′-GGGAATTCCATATGAAGGAGGAAAAAAACATGGACTTTCCGCAGCAACTC-3′) and IspA-R (5′-GGAAGATCTTTATTTATTACGCTGGATGATGT-3′) and *E. coli* BL21(DE3) genomic DNA as a template. The isolated *IspA* gene fragment was excised using BglII and NdeI, followed by insertion into the corresponding sites of the vector pYJM41 to create pYJM42.

**Figure 6:**
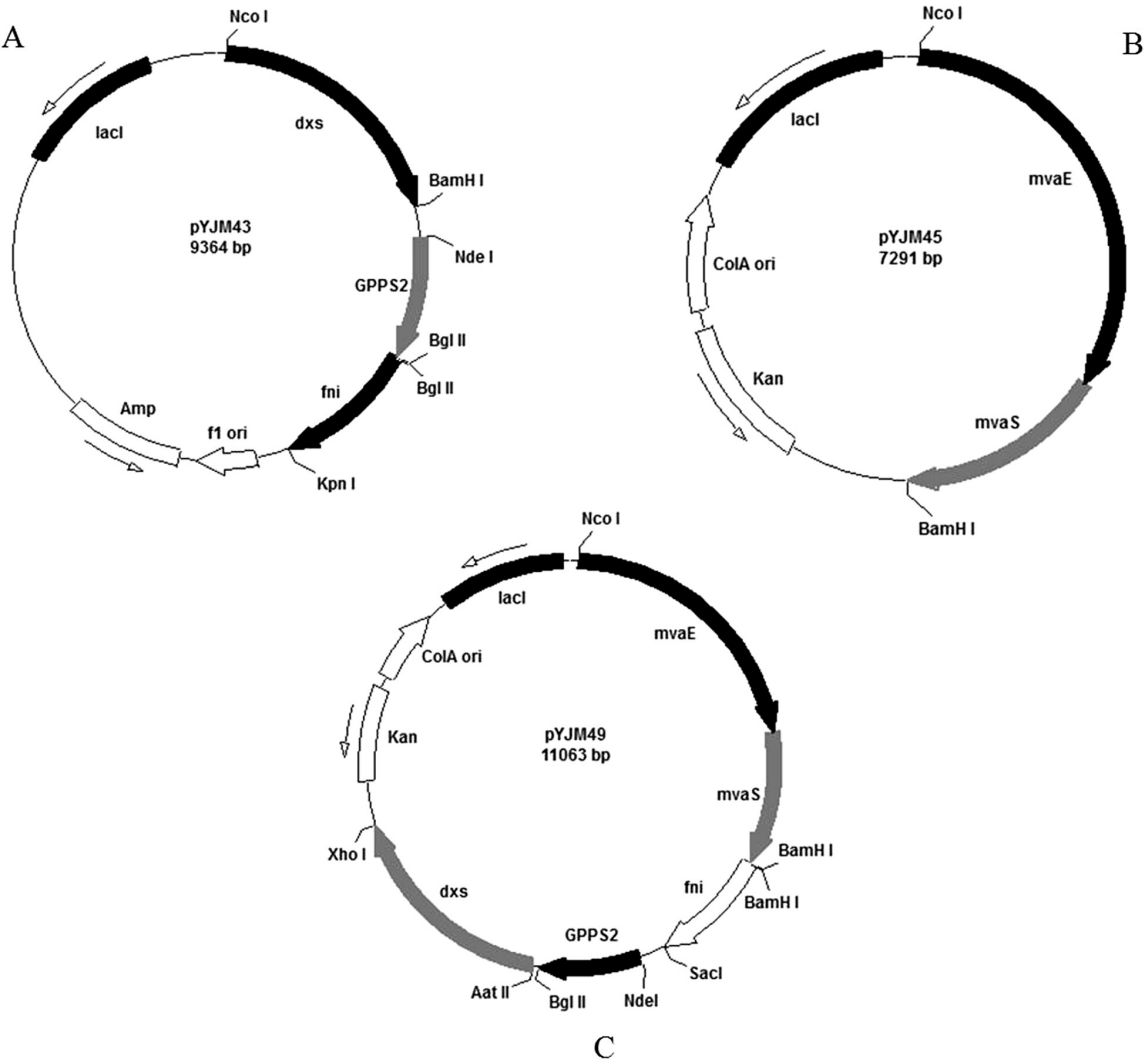
**Main plasmids used in this study.** **(A)** represented the plasmid pYJM43 harboring *dxs* and *fni* from *Bacillus subtilis*, *GPPS2* from *Abies grandis*; **(B)** represented the plasmid pYJM45 carrying *mvaE and mvaS* from *Enterococcus faecalis*; **(C)** represented the plasmid pYJM49 containing *mvaE and mvaS* from *Enterococcus faecalis*, *dxs* and *fni* from *Bacillus subtilis*, *GPPS2* from *Abies grandis*.

The geranyl diphosphate synthase (*GPPS2*) gene (GenBank No. AF513112) from *Abies grandis* was analyzed by online software (http://www.genscript.com/cgi-bin/tools/rare_codon_analysis) and optimized to the preferred codon usage of *E. coli* (http://www.jcat.de/). The codon-optimized *GPPS2* gene was synthesized by Genray Company with the plasmid pGH as the vector (named pGH/*GPPS2*). The *GPPS2* gene fragment was obtained by digestion of pGH/GPPS2 with NdeI and BglII and then ligated into the corresponding sites of pYJM41 to create pYJM43 (Figure 6A).

### Construction of plasmids for the MVA pathway of β-carotene synthesis

The *mvaE-mvaS* gene fragment was obtained by PCR using the primers mvaE-F (5′-CATGCCATGGAGGAGGTAAAAAAACAATGAAAACA-3′) and mvaE-R (5′- CGCGGATCCTTAGTTTCGATAAGAGCGAACGGT-3′) and plasmid pYJM20 [14] as a template. The purified *mvaE-mvaS* gene fragment was excised using NcoI and BamHI, followed by insertion into the corresponding sites of the vector pET-28a(+) or pCOLADuet-1 to create pYJM44 or pYJM45 (Figure 6B), respectively.

The *mvaE-mvaS-1* gene fragment was obtained by PCR using the primers mvaE -F1 (5′-CCGCTCGAGAGGAGGTAAAAAAACAATGAAAACA-3′) and mvaE-R1 (5′-GGAAGATCTTTAGTTTCGATAAGAGCGAACGGT-3′) and plasmid pYJM20 [14] as a template. The purified *mvaE-mvaS-1* gene fragment was digested by XhoI and BglII, followed by insertion into the corresponding sites of the vector pBAD18 to create pYJM46.

Plasmid pYJM14 was constructed based on pTrcHis2B by introducing *ERG8*, *ERG12*, *ERG19* and *IDI1* from *S. cerevisiae* [39].

The plasmid pAC-BETA, kindly provided by Dr Francis X. Cunningham Jr, was used to produce β-carotene. The plasmid pAC-BETA contains all of the genes required for the synthesis of β-carotene, including *crtE* [GGPP (geranylgeranyl pyrophosphate) synthase], *crtB* (phytoene synthase), *crtI* (phytoene desaturase) and *crtY* (lycopene cyclase) from *Erwinia herbicola* [40], and retains a chloramphenicol resistance gene.

### Construction of the plasmids for β-carotene synthesis with the MEP and MVA pathways

The *fni-1* gene was obtained by PCR using the primers fni-F1 (5′- CGCGGATCC AAGGAGATGACTCGAGCAGAACGAAAAAGA-3′) and fni-R1 (5′-CTAGGAG CTCTTATCGCACACTATAGCTTGA-3′) and *Bacillus subtilis* genomic DNA as a template. The isolated *fni-1* gene fragment was excised using BamHI and SacI, followed by insertion into the corresponding sites of the vector pYJM45 to create pYJM47.

The *GPPS2* gene fragment was obtained by digestion of pGH/GPPS2 with NdeI and BglII and was then ligated into the corresponding sites of pYJM47 to create pYJM48.

The *dxs-1* gene was obtained by PCR using the primers dxs-F1 (5′-CTAGGACGTCAAGGAGATGGATCTTTTATCAATACAGGACC-3′) and dxs-R1 (5′-CCGCTCGAGTCATGATCCAATTCCTTTGTGT-3′) and *Bacillus subtilis* genomic DNA as a template. The isolated *dxs-1* gene fragment was excised using AatII and XhoI, followed by insertion into the corresponding sites of the vector pYJM48 to create pYJM49 (Figure 6C).

### Measurement of β-carotene production

To quantify β-carotene production, β-carotene was extracted from *E. coli* as previously described [42]. Cells were harvested by centrifugation at 13,000 *g* for 3 min and washed once with sterile water. The cell pellet was then resuspended in acetone (1 mL) and incubated at 55°C for 15 min in the dark. Samples were then centrifuged at 14,000 rpm for 10 min, and the acetone supernatant containing β-carotene was transferred to a new tube. The β-carotene production in different engineered strains was analyzed using high performance liquid chromatography (Agilent Technologies Series 1200 system, Agilent, USA) with a UV/VIS detector at 454 nm using a Symmetry C18 column (250 mm × 4.6 mm, 5 mm, Waters, Milford, USA). Methanol, acetonitrile and dichloromethane (21:21:8) were used as the mobile phase at a flow rate of 1 mL/min at 30°C [2]. β-carotene (Cat.No. C4582, Sigma, USA) was used as the standard. The results represented the means ± S.D. of three independent experiments.

### Shake flask cultures

The strain was inoculated in either initial fermentation medium or optimized fermentation medium. When necessary, the cultures were supplemented with 100 mg/L ampicillin, 34 mg/L chloramphenicol and 50 μg/mL kanamycin. The cells were induced at OD_600_ = 0.6 with 0.25 mM IPTG and further incubated at 30°C. The β-carotene extraction and analysis were performed as previously described [2,42]. Optimization of the fermentation medium and process was shown in Additional file 1.

### Fed-batch fermentation

Fed-batch cultures were carried out in a Biostat B plus MO5L fermentor (Sartorius Stedim Biotech GmbH, Germany) containing 3 L of the optimized fermentation medium (Amp 100 μg/mL, Cm 34 μg/mL, Kan 50 μg/mL) as described above. The temperature was initially maintained at 37°C, and 34°C after induction. The pH was controlled at 7.0, and the dissolved oxygen (DO) concentration was maintained at 20% saturation. During the course of fermentation, 40% glycerol was fed at a rate of 3 g/L/h. The cells were induced at OD_600_ = 12 by adding 0.05 mM IPTG. IPTG was added every 8 h during the 80 h fermentation. Conversion efficiency (gram to gram) of glycerol to β-carotene was calculated with the following equation:

$$ \mathrm{Y}={\mathrm{G}}_{\mathrm{s}}/{\mathrm{G}}_{\mathrm{g}}\times 100\% $$

Where Y = conversion efficiency (gram to gram, 100%); G_s_ = weight of β-carotene (g); G_g_ = weight of glycerol (g).

## Additional file

Additional file 1:
**Optimization of fermentation process.**

## Abbreviations

DMAPP: Dimethylallyl pyrophosphate
IPP: Isopentenyl pyrophosphate
GPP: Geranyl diphosphate
MVA: Mevalonate
MEP: Methylerythritol 4-phosphate
IPTG: Isopropyl β-D-thiogalactoside
PCR: Polymerase chain reaction
