# Supplementary material for: Biosynthesis of β-carotene in engineered E. coli using the MEP and MVA pathways
Source: Microb Cell Fact. 2014 Nov 18;13:160. doi: 10.1186/s12934-014-0160-x (PMC4239400; doi:10.1186/s12934-014-0160-x)
Supplement: Additional file 1: — Optimization of fermentation process. [file 12934_2014_160_MOESM1_ESM.docx]

**Optimization of Fermentation Process**

Optimization of fermentation medium was carried out in shake-flask experiments in triplicate series of 50 ml of fermentation medium incubated with the strain YJM49. Amp (100 μg/mL), Cm (34 μg/mL) and Kan (50 μg/mL) were added when it was necessary. *E. coli* strains were cultured in the broth for initial production of β-carotene and incubated in a gyratory shaker incubator at 37°C and 180 rpm. When the OD_600_ reached 0.6-0.9, IPTG was added to a final concentration of 0.25 mM, and the culture was further incubated at 30°C for 24 h. The β-carotene extraction and analysis were performed as described previously [[2](#_ENREF_2),[18](#_ENREF_18)].

**Effect of Organic** [**Nitrogen Source**](http://www.iciba.com/nitrogen_source)

The shake-flask cultures were incubated in initial medium with different organic [nitrogen source](http://www.iciba.com/nitrogen_source)s (10 g/L): beef extract (solarbio), beef powder (MDBio, Inc), tryptone

(Beijing AoBoXing Bio-Tech Co., Ltd) or yeast extract (Beijing AoBoXing Bio-Tech Co., Ltd)) at the above-mentioned culture conditions, and the β-carotene products were detected.

**Effect of carbon source**

Carbon source is the main feedstock in fermentation. Therefore, the commonly used carbon sources (glucose and glycerol, 20 g/L) were screened in shake-flask with the nitrogen-optimized initial medium, at the above mentioned culture conditions.

**Effect of Induction Temperature**

The *E. coli* strain was inoculated in 50 ml of optimized fermentation medium and cultured at 37°C with shaking at 180 rpm. When the OD_600_ of the bacterial culture reached 0.6-0.9, the shake-flask cultures were incubated at different induction temperatures (25°C, 30°C, 34°C or 37°C) for 24 h in 0.25 mM IPTG, and the β-carotene products were assayed.

**Effect of IPTG Concentration**

The shake-flask culture was incubated in different inducer (IPTG) concentrations (0.05 mM, 0.1 mM, 0.25 mM, 0.5 mM or 1 mM) at the above-optimized temperature for 24 h, and the β-carotene products were measured.
